# Functional near-infrared spectroscopy for speech protocols: characterization of motion artifacts and guidelines for improving data analysis

**DOI:** 10.1117/1.NPh.7.1.015001

**Published:** 2020-01-10

**Authors:** Sergio L. Novi, Erin Roberts, Danielle Spagnuolo, Brianna M. Spilsbury, D’manda C. Price, Cara A. Imbalzano, Edwin Forero, Arjun G. Yodh, Glen M. Tellis, Cari M. Tellis, Rickson C. Mesquita

**Affiliations:** a University of Campinas, Institute of Physics, Campinas, São Paulo, Brazil; b Brazilian Institute of Neuroscience and Neurotechnology, Campinas, São Paulo, Brazil; c Misericordia University, Department of Speech-Language Pathology, Dallas, Pennsylvania, United States; d University of Pennsylvania, Department of Physics and Astronomy, Philadelphia, Pennsylvania, United States

**Keywords:** functional near-infrared spectroscopy, motion artifacts, speech protocols, jaw movement, temporal muscle, reading protocols

## Abstract

Monitoring speech tasks with functional near-infrared spectroscopy (fNIRS) enables investigation of speech production mechanisms and informs treatment strategies for speech-related disorders such as stuttering. Unfortunately, due to movement of the temporalis muscle, speech production can induce relative movement between probe optodes and skin. These movements generate motion artifacts during speech tasks. In practice, spurious hemodynamic responses in functional activation signals arise from lack of information about the consequences of speech-related motion artifacts, as well as from lack of standardized processing procedures for fNIRS signals during speech tasks. To this end, we characterize the effects of speech production on fNIRS signals, and we introduce a systematic analysis to ameliorate motion artifacts. The study measured 50 healthy subjects performing jaw movement (JM) tasks and found that JM produces two different patterns of motion artifacts in fNIRS. To remove these unwanted contributions, we validate a hybrid motion-correction algorithm based sequentially on spline interpolation and then wavelet filtering. We compared performance of the hybrid algorithm with standard algorithms based on spline interpolation only and wavelet decomposition only. The hybrid algorithm corrected 94% of the artifacts produced by JM, and it did not lead to spurious responses in the data. We also validated the hybrid algorithm during a reading task performed under two different conditions: reading aloud and reading silently. For both conditions, we observed significant cortical activation in brain regions related to reading. Moreover, when comparing the two conditions, good agreement of spatial and temporal activation patterns was found only when data were analyzed using the hybrid approach. Overall, the study demonstrates a standardized processing scheme for fNIRS data during speech protocols. The scheme decreases spurious responses and intersubject variability due to motion artifacts.

## 1 Introduction

Functional near-infrared spectroscopy (fNIRS) is a robust tool for measuring brain function.^1^^–^^8^ It probes the brain using the differential absorption of near-infrared (NIR) light by hemoglobin. Owing to features such as high temporal resolution, low cost, and portability, fNIRS is frequently chosen by researchers to investigate human populations ranging from neonates^9^^–^^24^ to patients with severe injuries.^2^^,^^25^^,^^26^^–^^31^ Moreover, fNIRS is useful during a variety of activities, even in unconstrained environments.^32^^–^^34^ In speech task applications, for example, fNIRS can potentially determine whether cerebral blood oxygenation changes in subjects with speech and language disabilities are different from those of normal subjects,^8^^,^^35^^–^^37^ and fNIRS could track motor learning during treatment of individuals with voice disorders.

Several advances in preprocessing of fNIRS data are needed to bring speech applications to fruition. One important fNIRS limitation concerns the contribution of extracortical layers to the fNIRS signal. Light interacts with superficial layers such as the scalp, in addition to the cortex. Thus, hemodynamic changes in superficial layers and/or global systemic changes in the brain can affect fNIRS measurements and can produce misleading results and interpretation.^38^^–^^45^ One approach to address this problem, which has been partially successful, is to add detectors close to the light sources (typically source–detector separations less than 1 cm). These source–detector pairs are predominantly sensitive to the layers above the cortex, and one can use their information to account for extracortical signal contributions.^40^^,^^46^ In speech protocols, for example, it is known that partial pressure of arterial ${\mathrm{CO}}_{2}$ (${\mathrm{PaCO}}_{2}$) varies during inner and outer speech tasks,^47^^–^^49^ and the data in the short-separation channels can help remove systemic physiological contributions that are simultaneously present in both extracortical and cortical tissues.^46^

Another confounding factor is the motion artifact, which can produce misleading hemodynamic data based on spurious fNIRS responses.^50^ Motion artifacts largely originate from relative movement between the subject’s head and the optodes and are generally characterized as spikes and/or baseline changes in the recorded light intensity.^51^^–^^53^ Several methods to correct the fNIRS signals for motion artifacts have been proposed.^51^^,^^54^^,^^55^ However, the fNIRS community still lacks the “standard,” general guidelines for properly removing motion artifacts from measurements.^53^ Progress is needed because motion artifacts can produce misleading results and conclusions.

The problem of motion artifacts is particularly critical for tasks involving speech, since the movement of the jaw is inherent to speech production and dramatically affects coupling between optodes and the scalp. During speech, the temporalis muscle is responsible for moving the jaw. It contracts and relaxes, moving from its resting position and producing motion artifacts in fNIRS data. These artifacts have been previously reported,^53^^,^^56^^,^^57^ but the processes by which they affect fNIRS signals are not fully understood. Jaw movement (JM) artifacts, in particular, represent the main application limitation for fNIRS investigations of the mechanisms underlying speech. The creation of a standard, well-validated, and robust method for correcting speech-related processing protocols will facilitate better investigation of speech production, improved understanding of brain mechanisms associated with communication disorders, and validation of treatment strategies for speech disorders.

In this work, we investigate the fNIRS responses in speech-related tasks. We hypothesize that jaw motion produces motion artifacts generated by tasks involving speech production, and we use the JM (only) task to characterize the effect that motion artifacts have on fNIRS signals during speech tasks. This protocol enabled us to validate a hybrid motion artifact algorithm for removing unwanted artifacts related to JM in speech during fNIRS measurements. The efficacy of the proposed algorithm was tested in reading tasks performed by 41 healthy subjects. In these studies, we compared subject fNIRS responses while reading aloud with the fNIRS responses in the same subjects when reading silently. We found no significant difference in both reading tasks “after” application of the correction procedure. Overall, the results demonstrate that the new correction algorithm can efficiently remove motion artifacts due to speech. This advance, in turn, should open new opportunities for fNIRS in functional protocols involving speech.

## 2 Materials and Methods

### 2.1 Subjects and Experimental Protocol

We acquired data from 52 healthy young volunteers (26 females) with ages ranging from 18 to 23 years [mean (standard deviation) = 20 years old (3)]. Data from one subject were discarded due to bad optical coupling in most of the locations measured. Of the remaining volunteers, 41 subjects (20 females) performed randomized block-designed paradigms with 10 repetitions of three distinct tasks: JM, reading aloud (RA), and silent reading (SR).

For the JM task, we instructed each subject to simply move their jaws for a period of 5 s followed by 5 s of rest. With this task, we aimed to isolate artifacts caused by JM during speech production. To further elucidate the impact of longer periods of JM in fNIRS recordings, a subcohort of 10 subjects (six females) performed the JM task for a period of 10 s followed by 20 s of rest. In the RA and the SR tasks, each subject read a small passage aloud and silently, respectively, for 10 s followed by 10 s of rest. The reading text was standardized at a third- or fourth-grade reading level. Thus, the subjects were not cognitively challenged. All instructions were presented on a computer screen, and the experimental protocol took ∼19  min. The experimental protocol was approved by the local ethical committee and was conducted in accordance with the Misericordia University Institutional Review Board policies, where the measurements were carried out, following the principles from Helsinki II Convention from 20 August 1947. All subjects provided written informed consent prior to data acquisition.

### 2.2 Functional Near-Infrared Spectroscopy Signal Acquisition

We acquired all fNIRS data with a commercial continuous-wave NIRS system (NIRScout, NIRx Medical Systems, New York). The optical probe contained 16 sources at two different wavelengths (LEDs centered at 760 and 850 nm, ∼15  mW light power emission for each) and 16 detectors. This configuration enabled the use of 32 source–detector combinations (i.e., channels) at 3 cm, and two source–detector pairs at 0.8 cm (short channels). Data were acquired at 7.8 Hz.

**Fig. 1:**
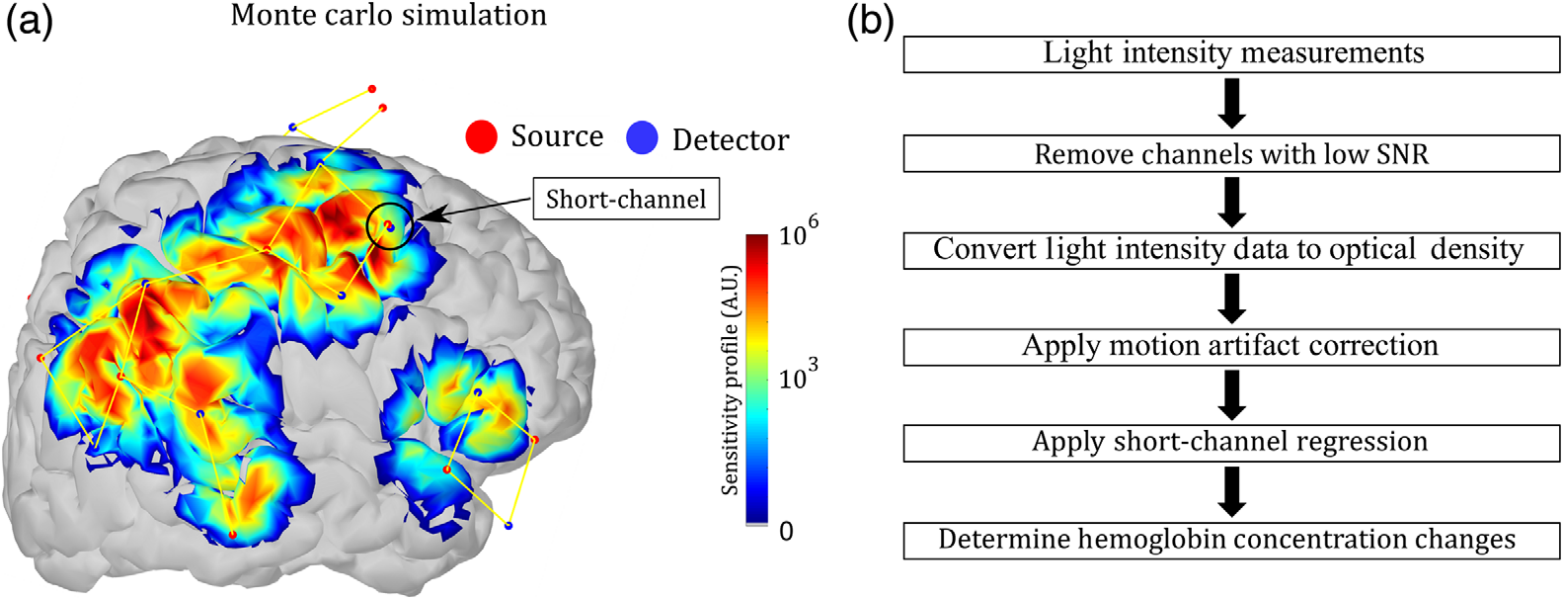
(a) Sensitivity profile of the optical probe on the right hemisphere. The probe was designed to cover most of the cortical regions related to speech, and it has a hemispherical symmetry. It employs 32 long-channels with source–detector separation of 3 cm, and two short-channels with source–detector separation of 0.8 cm. The short-channels were located in the frontal lobe (indicated in the figure). (b) Workflow of the preprocessing steps of fNIRS data.

The probe design was arranged to be sensitive to the primary regions related to language and speech in the frontal, temporal, and parietal lobes. We positioned one short channel in each brain hemisphere. The short-channel data enabled us to regress out extracortical contributions hemispherically. To secure the optodes on the heads of the subjects, we used a 10–20 standard cap from NIRx Medical Systems.^58^ We digitized the position of all optodes using a commercial digitizer (Fastrack, Polhemus, Colchester, Vermont) for better accuracy concerning the location of each source and detector. Figure 1(a) shows a sensitive profile for the probe, which was obtained from Monte Carlo simulations through the AtlasViewer package.^59^

### 2.3 Functional Near-Infrared Spectroscopy Data Analysis

To estimate hemoglobin concentration changes from fNIRS data, we wrote homemade scripts based on existing HomER 2 functions.^50^ The processing workflow is depicted in Fig. 1(b). First, we discarded channels with low signal-to-noise ratio (SNR) from the analysis. SNR was computed as the mean intensity of each channel divided by the standard deviation of the same channel. Our previous experience with fNIRS data found that channels with SNR <8 are very noisy and do not contain useful information.^12^^,^^13^^,^^30^ On the other hand, thresholds set higher than 8 sometimes discard good channels (i.e., channels that contain useful information).

Next, we converted light intensity from the remaining channels into optical density. Since we sought to understand the effects of motion at any frequency on the optical signal, we opted not to band-pass filter temporal fluctuations of the data for analysis. We then performed motion artifact correction using the optical density time series (see Sec. 2.4 about this motion artifact correction). After the motion artifact correction, we regressed out the extracortical contributions from superficial layers. Since superficial hemodynamics may not be homogeneously distributed along the scalp,^60^ the extracortical data were obtained from the short source–detector channel that was closest to a long separation channel of interest. This scheme minimized the effects of superficial hemodynamics. The regression procedure is described elsewhere.^40^^,^^42^^,^^44^^,^^46^ Hemoglobin concentration changes were calculated using the modified Beer–Lambert law with a differential pathlength factor of 6 for both wavelengths.^61^^,^^62^

For each channel in a single subject, we classified channels that presented typical hemodynamic responses due to neural activity using a general linear model with an adaptive hemodynamic function.^63^ The typical response was an increase in oxyhemoglobin and decrease in deoxyhemoglobin concentration synchronized with the period of stimulation. Channels that exhibited significant responses (p<0.05) for oxyhemoglobin and deoxyhemoglobin were considered to be activated during the task.^64^^–^^66^ To analyze the hemodynamic response function (HRF) of an activated channel, we block-averaged all trials of that channel.

### 2.4 Motion Artifact Correction

Current motion artifact correction algorithms can be classified into two main classes. The first class relies on first identifying time-series points that are contaminated by motion. These algorithms attempt to correct only the contaminated time points. The spline interpolation is probably the most well-known algorithm from this class. In this work, we used an automated version of the MARA algorithm for spline interpolation.^51^ Briefly, this spline algorithm identifies contaminated data segments by calculating the standard deviation within a sliding window. The segment correction is made by replacing the original data points with the residue of the data and a cubic spline interpolation. Baseline shifts were corrected by comparing the mean values of the data taken before and after the contaminated segment. More details regarding the spline interpolation algorithm can be found in Sec. S1 in the Supplementary Material.

The second class of algorithm includes those that correct the whole time series based on reliable features from the fNIRS data. Principal component analysis and wavelet decomposition are examples of algorithms that belong to this second class.^55^^,^^67^ In this work, we employed the wavelet decomposition analysis to correct the motion artifacts, since this algorithm has been used extensively already to process fNIRS data.^53^ In wavelet decomposition analysis, each fNIRS time series is decomposed in functions (i.e., wavelets) that are localized in both time and frequency.^55^ A correction filter sets wavelet coefficients with values very far outside the central portion of the distribution of coefficients to zero. The reasoning behind this procedure is that outlier coefficients are related to motion. The time series is then reconstructed without motion (see Sec. S2 in the Supplementary Material, for more details about wavelet filtering).

We tested several approaches to perform motion artifact correction. Specifically, we analyzed the efficacy of one algorithm from each class to independently remove speech-related artifacts generated by JM. However, since these algorithms are based on different hypotheses, we further analyzed the effects of a hybrid motion artifact correction algorithm that “sequentially” uses spline and then wavelet decomposition, “in this order.” By employing this hybrid algorithm, we showed that prior spline correction can improve the performance of subsequent wavelet analysis per motion artifact removal.

### 2.5 Statistical Analysis

To extract common activation patterns from the whole group for each task (RA and SR), we performed a full-frequency analysis across all subjects rather than just group averages. Specifically, channels with (without) the characteristic HRF were assigned with 1 (0) so that we could create binarized classification vectors (BCVs) for each subject. Next, we computed frequency maps for each task by combining all BCVs. Here, we opt to present group results with frequency analysis, because the individual response to functional tasks is very heterogeneous, and thus simple averages do not reflect the whole distribution of responses measured across a group. In addition, the frequency analysis enables one to find the cortical regions that are mostly present in all subjects for a given task, which we have previously shown to be more informative than average property maps; the average property maps are not always representative of individual responses when the distribution of responses is wide across subjects.^12^^,^^13^^,^^68^ Finally, our summaries of the activated patterns related to tasks used three regions of interest (ROIs) known to play a role during reading: Broca’s area; Wernicke’s area; and the posterior region in the right hemisphere, contralateral to Wernicke’s area. The channels in each ROI were manually chosen based on their anatomical position. Owing to lack of accurate spatial information, we averaged the results of the activated channels across all subjects.

## 3 Results

### 3.1 Jaw Movement Induces Two Types of Motion Artifacts

We visually inspected the optical density time series of each channel for each subject during the JM (only) task. We found that movement of the jaw produces strong (apparent) changes at both wavelengths that are temporally correlated with task duration. These changes are easy to be incorrectly assigned as brain activation (i.e., unreal brain activation) in any functional speech task. Concerning location, the changes are broadly distributed over the different regions of the brain related to speech production. Many (a majority of) channels are compromised. Overall, the mean (standard deviation) number of contaminated channels across all subjects was 20 (5), which corresponds to 60% of channels in our optical probe.

**Fig. 2:**
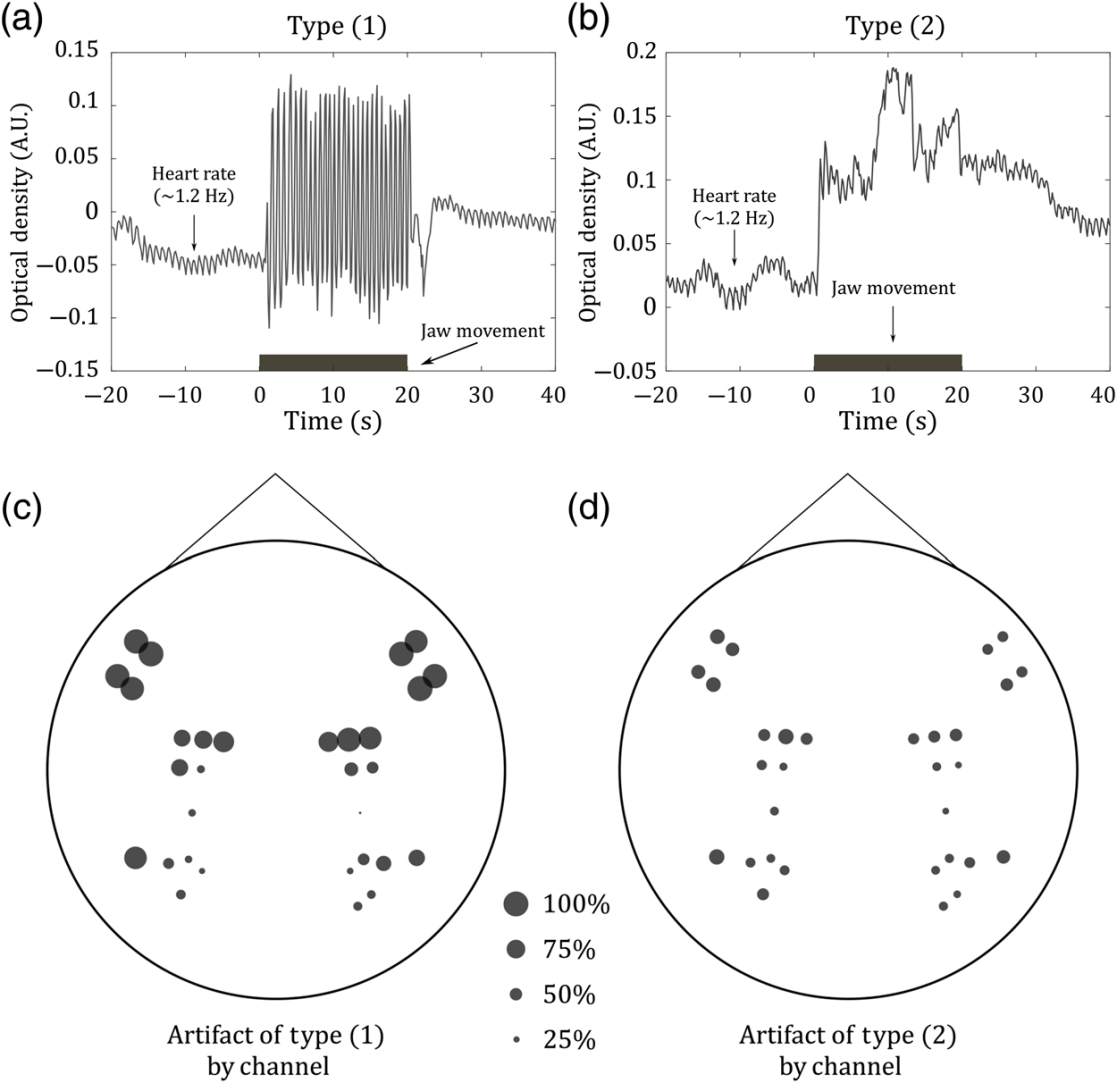
Temporal and spatial characterization of motion artifacts due to the movement of the jaw. (a) and (b) Arbitrary data affected by abrupt, nonharmonic oscillations (type 1 artifact) and by a significant change in baseline temporally correlated with the jaw motion (type 2 artifact), respectively. (c) and (d) The spatial distribution of the artifacts of type 1 and type 2, respectively, across all subjects. In the figure, the circles represent the channels, and the size of the circle represents the how frequent the jaw motion artifact was detected in these positions across the cohort. (Note, the 100% size, etc., is indicated outside the figure for reference.) The location of each channel was estimated as the average position (from a two-dimensional projection of our probe) of each channel’s source and detector.

The manner in which each channel was affected by JM was not similar. JM primarily induced two types of artifacts in the fNIRS data. The first type (type 1) is characterized by abrupt, nonharmonic oscillations synchronized with the duration of the stimulus [Fig. 2(a)]. The second type of artifact (type 2) introduces baseline changes distributed within the stimulus period. They are similar to motion artifacts commonly found in other functional tasks [Fig. 2(b)]. Type 1 artifacts are present in the short- and long-duration JMs, but artifacts of type 2 become more important as the duration length of the speech task increases. Concerning location, type 1 artifacts mostly affect channels located in the anterior region of the head, i.e., closer to the main parts of the temporalis muscle [Fig. 2(c)]. Type 2 artifacts were most commonly found in posterior channels [Fig. 2(d)]. Some channels, mostly those located between anterior and posterior regions, exhibit both types of artifacts: abrupt oscillations and slower baseline changes.

### 3.2 Hybrid Procedure of Wavelet and Spline Removes Artifacts Caused by Jaw Motion

**Fig. 3:**
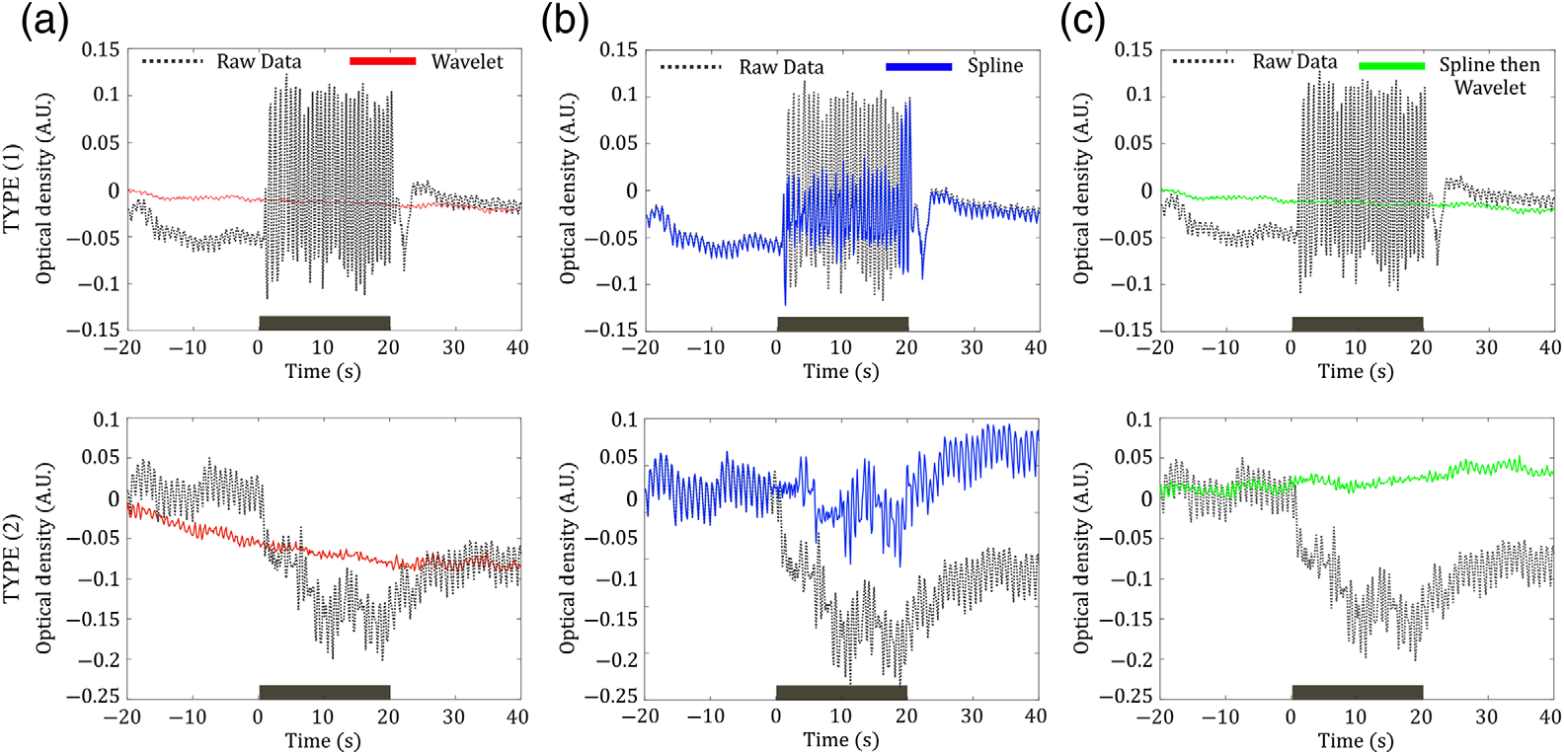
Performance of motion artifact removal in decontaminating (“removing/fixing”) JM artifacts in one illustrative subject. Each algorithm was tested separately in both type 1 (top row) and type 2 (bottom row) artifacts. In all plots, the gray line represents the raw optical density (760 nm) time series for a representative channel. Red, blue, and green colors correspond, respectively, to the optical time series after motion correcting with wavelet decomposition, spline interpolation, and the hybrid algorithm (i.e., spline interpolation followed by wavelet decomposition). (a) and (b) The performance of wavelet decomposition and spline interpolation, respectively, on motion artifacts. (c) The performance of our hybrid procedure for correcting motion artifacts.

Since JM is not expected to induce a significant average response variation, the signal mean of the time points immediately before the task should be approximately the same as the mean of the time points during the JM task (note, participants did not read or speak during the JM tasks; they only moved their jaw). However, this effect is not what one observes. We attempted to apply fNIRS motion artifact correction algorithms to remove such artifacts. Across all JM trials for all subjects, wavelet filtering was able to correct ∼90% of the trials during the JM task (i.e., corrected data imply that the mean of the time points during the JM task was not significantly different from the mean of the time points immediately before the JM task). The wavelet decomposition could successfully remove abrupt oscillations in the optical density time series, but it did not succeed in correcting the artifacts of type 2. Instead, the wavelet algorithm left/induced long-term trends in intervals of the time series that exhibit baseline changes [Fig. 3(a)].

On the other hand, spline interpolation is very efficient for correcting type 2 artifacts, which are characterized by baseline changes. Spline-only, however, could not remove the abrupt changes in concentration, which characterize JM type 1 artifacts [Fig. 3(b)]. This failure is likely related to the poor performance of the algorithm in locating the artifacts. Specifically, the spline algorithm relies on automatically identifying points that are contaminated by motion artifacts. This identification is based on abrupt changes in signal intensity. Since JM promotes consistent abrupt oscillations, the automated detection fails to identify the artifacts, because it starts to treat the oscillations as a reliable feature. Type 1 artifacts are more frequent than type 2; therefore, the performance of the spline algorithm “alone” to correct all trials was very poor. Alone, the spline algorithm properly corrected only 10% of all the trials affected by motion.

Since the performance of wavelet and spline was complementary (the 10% spline success is possibly a subset of the 10% of data that the wavelet could not correct), we hypothesized that the most efficient approach to remove artifacts in speech tasks would be to combine the spline and the wavelet algorithms. To this end, first, the spline algorithm interpolation is used to correct baseline changes, since the wavelet decomposition does not properly handle baseline changes (it can induce data trends). Wavelet decomposition is applied to the data after the spline algorithm is complete. Figure 3(c) shows the effect of our proposed approach in a representative time series around the JM task. Compared to wavelet filtering only, the hybrid procedure has a positive impact on correcting JM artifacts of type 2, since it removes artificial data trends, while it does not yield any negative effect on JM artifacts of type 1. The improvement of performance on type 2 artifacts generates a better rate of corrected trials, i.e., the hybrid approach corrected ∼94% of the data across all subjects without creating long-term trends.

### 3.3 Validation of the Proposed Algorithm in Reading Tasks

Lastly, we validated our approach in an experimental protocol of healthy subjects during reading tasks. We hypothesized that the method of reading (RA versus SR) should not change the functional activation pattern measured by fNIRS during the task. That is, after JM is factored out, the functional activation responses should be similar for RA and SR. Figure S1 in the Supplementary Material shows a representative example of how the motion of the anterior temporal muscle affects the optical signal during a RA trial.

Our first observation is that a robust method for correcting motion artifacts saves more data for analysis. Without correcting the motion artifacts during the RA task, for example, ∼76% of the subjects (31/41) would have been discarded because they did not present any channel with characteristic hemodynamic response. This situation changes after correcting for motion artifacts. When applying the wavelet decomposition algorithm only and the hybrid algorithm, respectively, we were able to analyze data from 18 and 23 subjects, respectively, which represent 43% and 56% of the total cohort, respectively.

**Fig. 4:**
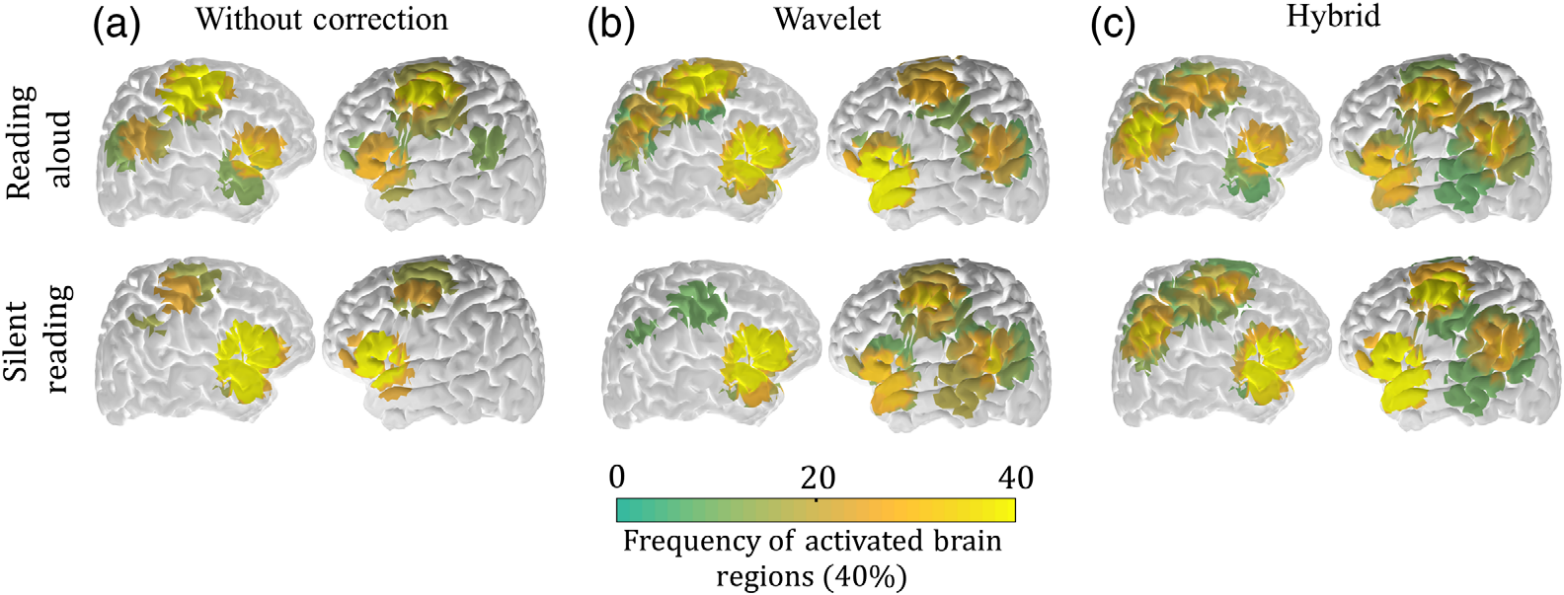
Comparison between activated brain regions from RA (first row) and SR (second row) as a function of processing of motion artifacts: (a) without any motion artifact correction, (b) after using wavelet only correction, and (c) after using hybrid procedure to remove motion artifacts. Each brain region is colored based on the frequency of activation across the group, considering only subjects that had at least one activated channel. Channels that presented characteristic hemodynamic response for oxy-hemoglobin (HbO) and deoxy-hemoglobin (HbR) were considered to be activated. From the plots it is possible to notice that the hybrid method yields the closest match between the two tasks, both qualitatively (i.e., the regions activated) and quantitatively (i.e., the frequency of activation of each region). The wavelet-only correction underestimates the activated areas in the parietal regions in both hemispheres.

**Fig. 5:**
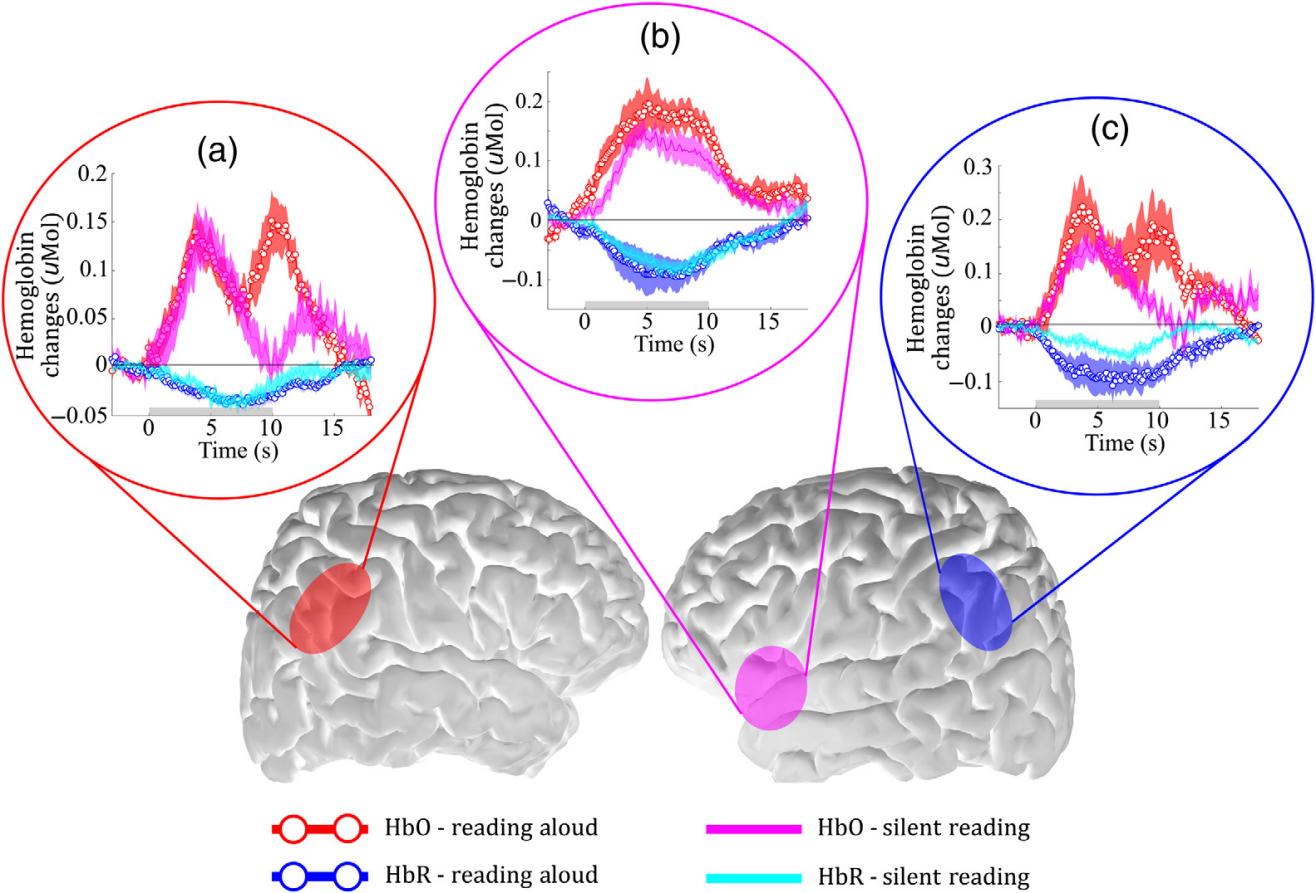
Temporal dynamics of the HRF due to reading tasks across all subjects from three different ROIs: (a) posterior region in the right hemisphere (contralateral to Wernicke’s), (b) Broca’s area, and (c) Wernicke’s area (left hemisphere) after applying our hybrid method to remove motion artifact. The gray bar on the plots indicates when the task was performed, and the shaded error bars are the standard error across all channels in the ROI. All ROIs are localized in the brain to facilitate visualization.

The group response during RA and SR is shown in Fig. 5 for the three cases considered already (no motion artifact correction, wavelet correction only, and hybrid approach). When motion artifact correction was applied, Broca’s and Wernicke’s areas had a relatively high frequency in both tasks, as is expected for reading tasks. However, the activated regions are more similar between the two reading tasks after correcting motion artifacts with the hybrid approach [Fig. 4(c)]. It is also interesting to note that the motion artifact correction was even important for improving the response of the SR task. This improvement is expected, since JM is not the only source of motion artifact in fNIRS data.

We compared the temporal dynamics of the HRF for both reading tasks after using the hybrid approach for motion artifact correction. Notice the hybrid algorithm provided the highest spatial agreement between the activated regions due to RA and SR (Fig. 5). In all activated ROIs, we observed excellent agreement between the averaged HbO and HbR. The Pearson’s correlation coefficients for the curves obtained during RA versus the curves obtained during SR ranged from 0.53 to 0.96, again demonstrating that the hybrid algorithm provides robust results both in the area of activation and in the dynamics of the response measured. Interestingly, by comparing the average HRF of both tasks it is possible to see that RA and reading silently induces changes with similar amplitudes in all ROIs analyzed. Typically, motion artifacts induced by the temporal muscle increase HbO and HbR changes by 10-fold.^57^ The similarity in amplitude that we found in the two reading tasks further suggests that the artifacts due to the temporalis muscle were properly corrected with our hybrid algorithm.

## 4 Discussion

Owing to its high portability and ease of use, fNIRS is attractive for measurement of brain activation in functional protocols involving speech.^8^^,^^36^^,^^37^^,^^69^ The fNIRS time-series data, however, must be carefully analyzed and interpreted since the technique is sensitive to confounding factors such as motion artifacts and extracortical hemodynamic changes.^38^^–^^43^^,^^44^^,^^45^^,^^51^^,^^55^^,^^68^^,^^70^^–^^73^ Despite recent efforts to standardize fNIRS processing workflow, certain applications of fNIRS may need unique methods to deal with inherent artifacts underlying a specific task and/or experimental protocol. In this work, we aimed to investigate this issue per use of fNIRS in speech-related protocols. We demonstrated a method for data analysis of such functional protocols that is readily standardized.

In many speech-related tasks, the motion of the temporalis muscle (associated with JM) can lead to unique motion artifacts.^56^ In this work, we attempted to characterize the artifacts in speech by performing fNIRS measurements during JM. We observed that JM produces two main types of motion artifacts (Fig. 2): abrupt, nonharmonic oscillations specific to voice production (type 1) and baseline changes (type 2). While type 1 artifacts greatly affect most of the channels located in the frontal and temporal lobes, type 2 artifacts were found in more posterior channels and played a major role in long-duration tasks [Fig. 2(c)]. Type 2 artifacts are commonly reported as motion artifacts in any fNIRS study, but type 1 artifacts have been under-reported because previous studies largely used band-pass filtering in fNIRS analysis. The use of low-pass filters smooths out the oscillation pattern but leaves signatures at the same frequencies of the task-induced changes. Importantly, since these artifacts are correlated with the task, they can lead to significant hemoglobin variation during the task, i.e., if not corrected properly.

To properly correct fNIRS data for speech protocols, we demonstrated a hybrid motion artifact removal algorithm by combining two complementary methods: spline interpolation and wavelet decomposition, in this order. This approach has been recently suggested to correct motion artifacts produced in infant fNIRS data,^74^ and our work extends its use to another protocol that is also affected by motion. Moreover, the hybrid approach is based on the expectation that spline interpolation can efficiently correct baseline changes (type 2 artifacts) but fails to remove abrupt oscillations due to the motion of the jaw (type 1 artifacts). Wavelet regression alone could remove JM artifacts efficiently, but in practice it created long-term trends and skewed the data [Fig. 3(a)]. This limitation of wavelet correction is in agreement with previous reports.^55^^,^^75^

As an aside, we compared the performance of the hybrid method with the correlation-based signal improvement (CBSI) approach,^54^ which has been noted as an alternative method for analyzing tasks involving language protocols.^36^^,^^53^ With our JM data, the hybrid method performed better than the CBSI algorithm (results not shown). CBSI could only fully correct 12 of 41 subjects. More importantly, three subjects had more than 10 uncorrected channels by the CBSI. Both the wavelet-only and the hybrid algorithms had 0 subjects with more than 10 uncorrected channels. This poor performance of the CBSI method in real data is in agreement with previous reports.^53^^,^^75^ In addition, we believe that our approach is less limited than CBSI, since it does not force anticorrelations between oxyhemoglobin and deoxyhemoglobin concentration changes. Note the opposite behavior between HbO and HbR is not necessarily expected in all functional tasks,^49^^,^^76^ and even when it occurs it may not occur at the exact same time. The high temporal resolution of NIRS may lead to spurious correlations as well.^77^

Finally, the hybrid approach was successfully validated in a functional protocol involving reading. We chose this task because reading can be performed both silently and out loud. Therefore, we could analyze the influence of speech in the protocol alone by keeping all other cofactors constant. Our results show that both tasks activate the main areas related to reading, including Broca’s and Wernicke’s regions (Fig. 4), despite high intersubject variation (as expected for cognitive or higher-processing brain function). Figure 5 compares the average HRF from the two reading tasks measured across all subjects in the main activated regions. The robust similarity of the amplitudes and dynamics of the curves in each brain region suggests that contributions of motion artifacts due to speech were removed.

Despite this effort to remove unwanted contributions from extracortical layers and superficial task-evoked artifacts from systemic physiology, our short-channel regression approach has limitations. First, due to experimental constraints, we limited the number of short separation channels to two. Previous studies provided evidence that the superficial hemodynamics is not homogenous across the different regions of the brain,^60^^,^^78^ and therefore the improvement obtained by using two short separation channels decreases as the distance between the short and the long channels increases.^79^ Unfortunately, most of the studies that report the task-evoked effects of ${\mathrm{CO}}_{2}$ in speech were not performed with short separations (i.e., <1  cm);^47^^,^^48^ therefore, it is not possible to know how much of these ${\mathrm{CO}}_{2}$ effects are from the cortex, and we cannot point whether these effects are still present in some of our channels after short-channel regression. Regardless, we acknowledge that the known task-evoked effects of ${\mathrm{CO}}_{2}$, as well as effects from systemic low-frequency oscillations, might not have been completely eliminated. This would affect mainly the channels furthest away from the short-separation channels. Overall, despite these limitations mentioned, the (temporal and spatial) comparison between the two reading tasks strongly suggests that our proposed workflow removes spurious hemodynamic response in speech and decreases the variability of the hemodynamic response measured in speech-related protocols.

## 5 Conclusions

In this work, we characterized the effects of speech production on the fNIRS signal and developed an approach to ameliorate motion artifacts. We performed a JM task in 50 healthy subjects to characterize the motion artifacts induced by speech, and we observed that JM induces two distinct patterns of motion artifacts in the fNIRS signal. To remove unwanted contributions, we validated a hybrid motion-correction algorithm based, sequentially, on spline interpolation and then wavelet filtering. Finally, we validated the hybrid algorithm in 41 healthy subjects during a reading task performed under two different conditions: RA and SR. After applying the hybrid procedure, we observed good agreement between both tasks, both spatially and temporally. Overall, this study demonstrates a standard processing scheme for fNIRS data during speech protocols that decreases spurious responses and intersubject variability due to motion artifacts.
