# Supplementary material for: Functional near-infrared spectroscopy for speech protocols: characterization of motion artifacts and guidelines for improving data analysis
Source: Neurophotonics. 2020 Jan 10;7(1):015001. doi: 10.1117/1.NPh.7.1.015001 (PMC6953699; doi:10.1117/1.NPh.7.1.015001)
Supplement: Supplementary file 1 [file NPh_007_015001_SD001.pdf]

Functional near-infrared spectroscopy for speech protocols: characterization of motion artifacts and guidelines for improving data analysis

S.L. Novi[1,2], E. Roberts[3], D. Spagnuolo[3], B.M. Spilsbury[3], D.C. Price[3], C.A. Imbalzano[3], E. Forero[1,2], A. G. Yodh[4], G.M. Tellis[3], C.M. Tellis[3], R.C. Mesquita[1,2]

[1] Institute of Physics, University of Campinas, Campinas, SP, Brazil 13083-859

[2] Brazilian Institute of Neuroscience and Neurotechnology, Campinas, SP, Brazil 13083-888

[3] Department of Speech-Language Pathology, Misericordia University, Dallas, PA 18612

[4] Department of Physics & Astronomy, University of Pennsylvania, Philadelphia, PA 19104

Address for Correspondence: Rickson C. Mesquita
DRCC/IFGW
R. Sergio Buarque de Holanda, 777
Campinas, SP (Brazil) 13083-859
Phone Number: +55 (19) 3521-0137
Fax: +55 (19) 3521-5512
Email: rickson@ifi.unicamp.br

SUPPLEMENTARY MATERIAL

S1 - Spline interpolation

The spline interpolation algorithm used in this work is based on the MARA algorithm introduced by Scholckmann et al.[51] In summary, the algorithm splits the fNIRS data of each channel into data segments and computes the standard deviation in each segment. The contaminated data segments by motion artifacts are identified based on the moving standard deviation (S(t)) given by:

$$S(t) = \left(\frac{1}{2k+1}\left[\sum_{j=-k}^{k} x^2(t+j) - \frac{1}{2k+1}\left(\sum_{j=-k}^{k} x(t+j)\right)^2\right]\right)^{\frac{1}{2}} \quad (1)$$

for $t = k+1, k+2, \ldots, N-k$. Here, N is the length of the time series, and *2k +1* is the sliding window length. k is a free parameter that defines the length of the segment and must be set *a priori*. By empirically investigating the impact of k on the performance of MARA in our data, we concluded that one efficient way to define k is to set $k = 2.5 \times f_a$ where f_a is the acquisition frequency of the fNIRS time-series. By doing that, fluctuations due to heartbeat are not interpreted as motion artifacts.

After computing the moving standard deviation, one has to define an absolute threshold for identifying data segments that are contaminated by motion artifacts. Note that contaminated segments have higher values of S due to abrupt changes. Next, the identified segments are modeled by a cubic spline interpolation, and the contaminated time points are replaced by the result of the subtraction of the original time series by the interpolated data. Finally, the algorithm corrects the baseline shifts by properly adding values in each data segment based on comparisons between the mean value of the segments before and after the contaminated segment.

The main drawback of the MARA algorithm is that S(t) depends on the signal-to-noise ratio (SNR) of each channel. In our data cohort, we verified that it is hard to find an optimal threshold that suits every channel. To overcome this limitation one can define one threshold for each channel of each subject. Since in this work we applied the spline interpolation algorithm in the optical density time-series, this proposed procedure would lead to the definition of two threshold values per channel (one per wavelength). This process, however, is time-consuming and subjective.

In order to avoid the necessity of defining several thresholds, we defined the threshold for each channel based on the distribution of the values of the moving standard deviation for each optical density time-series. In fact, we defined the threshold of each time series as the mean value of S summed with a multiple of the standard deviation of S. To clarify, let δ_i be the standard deviation of S for the *i*th optical density time-series (OD_i). Thus, the threshold value (R_i) of OD_i will be given by $R_i = \gamma\delta_i$ in which γ is a free parameter that needs to be defined by the user. For

the present study, we set $\gamma = 0.9$ after testing several values. We define γ in such a way that the spline interpolation was optimized to be more sensitive to baseline changes than to motion-induced spikes. Another important change in the algorithm is that we decided to apply the spline correction only in the block period (baseline + stimulus + recovery period) instead of in the whole time series. We have observed that this procedure is helpful in long data cohorts with different tasks or long rest and baseline periods because different data conditions usually have different degrees of susceptibility to motion artifacts.

S2 – Wavelet decomposition

To perform the wavelet motion artifact removal proposed by Molavi et al.,[55] we used the *hmrMotionCorrectWavelet* MatLab function available in Homer2[50]. The wavelet-based motion artifact removal algorithm performs motion artifact corrections individually for each channel and wavelength. The algorithm assumes that the recorded NIRS data ($y(t)$) is composed of real-physiological signals, $f(t)$, and spurious signals, $\varepsilon(t)$, which are originated from motion artifacts. The main idea behind wavelet filtering is similar to applying a band-pass filtering via a Fourier transform. However, in wavelet filtering, the signal is decomposed in wavelets, which are functions that are well localized in both time and frequency[52]. (Note, for comparison in Fourier decomposition the signal is decomposed in sins or cosines, which are highly localized in frequency, only.) After decomposing the recorded signal with the discrete wavelet transform (DWT), wavelet coefficients that have a higher probability of belonging to motion artifacts than to physiological data are set to zero. Next, the signal is reconstructed via the inverse wavelet transform. In principle, the removal of coefficients should be easy since motion and physiological data should have considerably different signatures in the wavelet domain.

The sensible step of wavelet filtering is to define the probability threshold for removing wavelet coefficients that come from motion artifacts. Compared to motion artifacts, the hemodynamic signal is smoother, presenting slower variations. Hence, we can reasonably assume that the distribution of wavelet coefficients from the physiological data should have a normal distribution with smaller variance compared to artifact coefficients. In addition, the coefficients are spread around zero because they are outputs of a high-pass filter. In this sense, the model proposed by Molavi et al. assumes that the wavelet coefficients of each wavelet decomposition level can be written as $w = w' + \lambda$, where w' is a normal distribution centered at zero, and the λ coefficients are a few large outliers from the whole distribution w due to motion artifacts. To decide whether a coefficient comes from motion, we set an arbitrary probability α such that any coefficient w is set to zero if its probability of occurring is less than α. In the present work, we used $\alpha = 0.1$ based on previous works.[52,53]

Supplementary Figures.

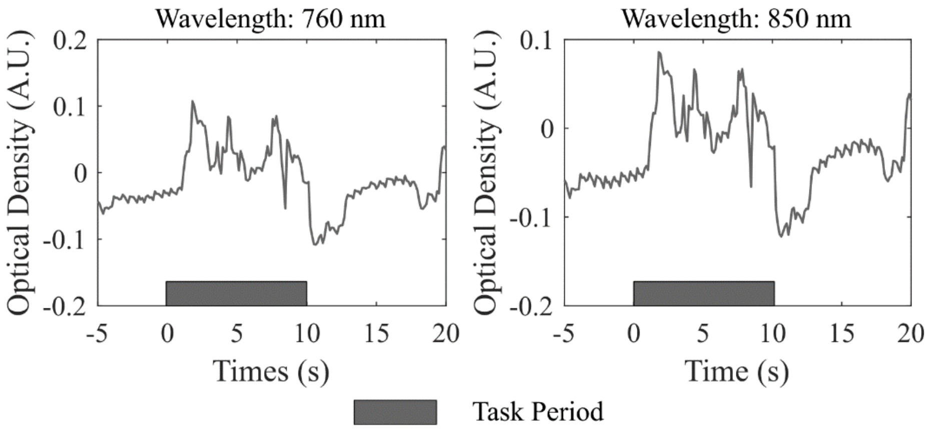

Figure S1. Optical density for both wavelengths for one representative channel of a single subject during one trial of the “Reading Aloud” task.
